# Case report: Sarcomatoid renal cell carcinoma masquerading as hydronephrosis

**DOI:** 10.3389/fonc.2024.1361250

**Published:** 2024-05-22

**Authors:** Shicheng Chen, Zhongcong He, Mi Meng, Zhong Tian, Cheng Zhu, Ni Fu, Bo Yu

**Affiliations:** ^1^ Department of Urology, the Second Affiliated Hospital of ZunYi Medical University, Zunyi, China; ^2^ Department of Thoracic Oncology, the Second Affiliated Hospital of ZunYi Medical University, Zunyi, China

**Keywords:** misdiagnosis, rare disease, aggressive renal cell carcinoma, hydronephrosis, sarcomatoid renal cell carcinoma

## Abstract

Sarcomatoid renal cell carcinoma (SRCC), a manifestation of sarcomatoid dedifferentiation in renal cell carcinoma, is characterized by elevated invasiveness and a grim prognosis. Typically, SRCC patients present with advanced or metastatic conditions and survival rates rarely extend beyond one year. In this study, we describe a case of SRCC characterized by the patient exhibiting right flank pain without hematuria. Initially, imaging interpretations led to a diagnosis of severe hydronephrosis. Subsequently, an open right nephrectomy post-surgery confirmed the pathology of sarcomatoid renal cell carcinoma.

## Introduction

Renal cell carcinoma (RCC) is recognized as one of the most lethal malignancies affecting the urinary system (1). Sarcomatoid renal cell carcinoma (SRCC), which constitutes a mere 5% of RCC cases, represents a highly resistant and deadly manifestation of kidney cancer (2). Patients diagnosed with SRCC commonly present advanced or metastatic conditions, resulting in an exceptionally dismal prognosis (3). The absence of distinct clinical symptoms and imaging characteristics can contribute to delayed diagnoses or misinterpretations. The occurrence of sarcomatoid renal cell carcinoma masquerading as severe renal hydronephrosis is exceedingly uncommon. In this report, we detail a clinical case that exemplifies this rarity. The patient initially sought medical attention due to persistent lumbar pain of unidentified origin, which led to an initial diagnosis of severe right renal hydronephrosis based on imaging findings. Consequently, an open right nephrectomy was performed, unveiling sarcomatoid renal cell carcinoma through postoperative pathology.

## Case report

**Figure 1:**
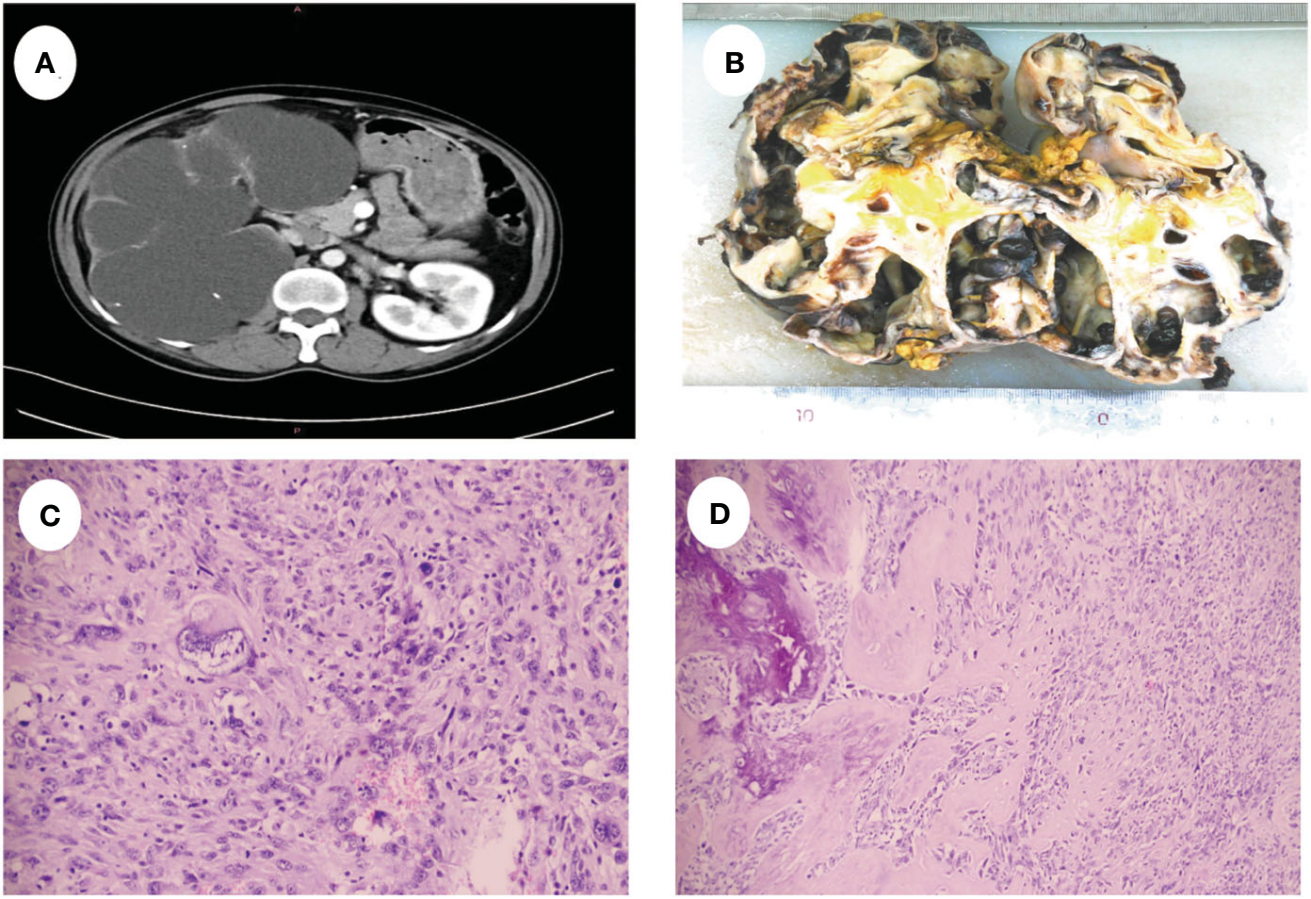
**(A)** CT shows severe hydronephrosis of the right kidney. **(B)** Shows the gross appearance of the right kidney, the size was about 20cm x 15cm. **(C)** The tumor cells were disorganized and showed sarcomatoid changes with marked heterogeneity. **(D)** Tumor cells are spindle-shaped with ossification formation.

**Figure 2:**
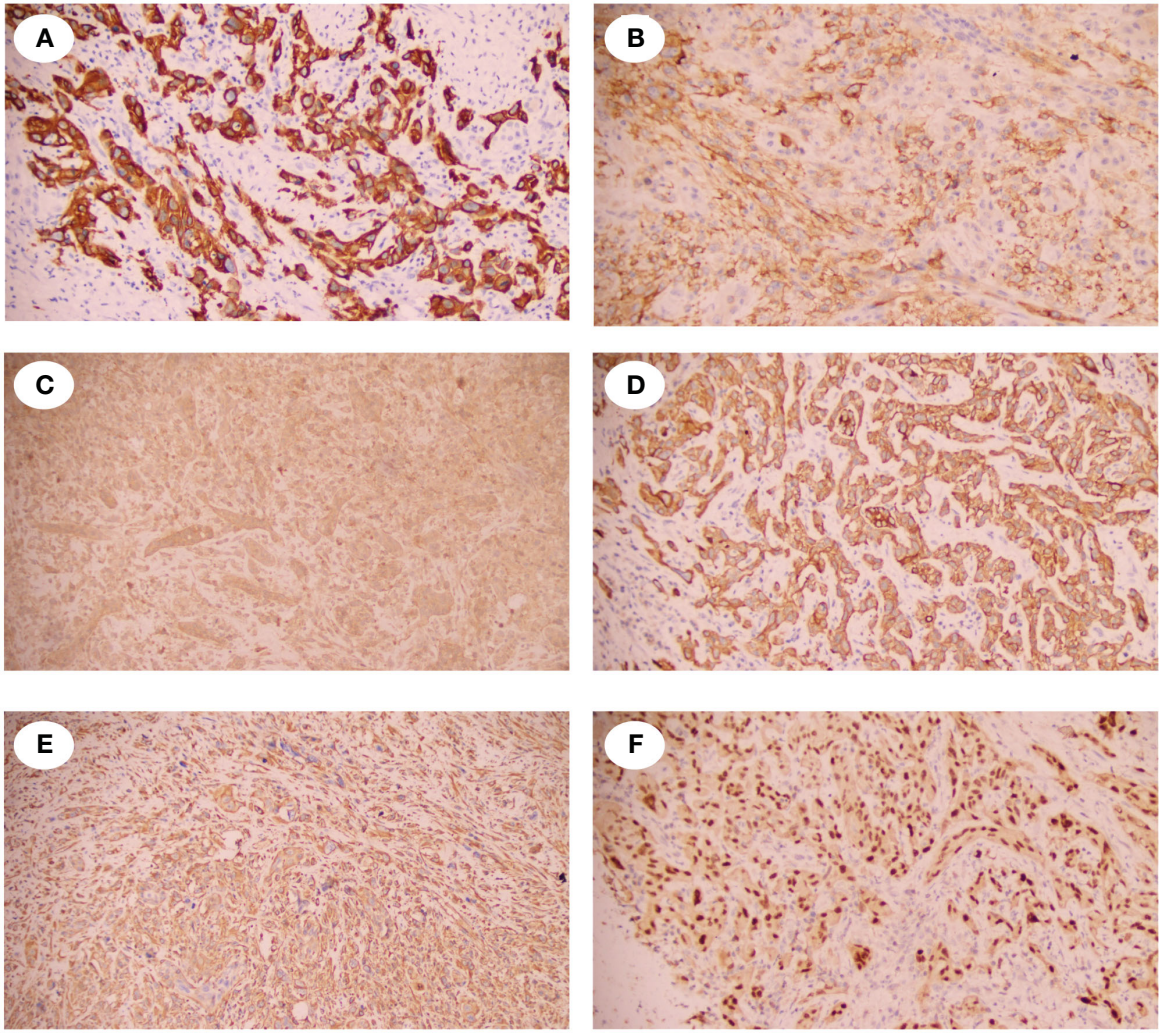
Immunohistochemical staining of the SRCC. **(A)** showed the spindle tumor cells were cytoplasmic positive for CD5/6. **(B)** showed the spindle tumor cells were cytoplasmic positive for CD10. **(C)** showed the spindle tumor cells were cytoplasmic positive for CD99. **(D)** showed the spindle tumor cells were cytoplasmic positive for CK. **(E)** showed the spindle tumor cells were cytoplasmic positive for Vinentin. **(F)** showed the spindle tumor cells were nuclear positive for PAX-8.

A 52-year-old male presented with recurrent lumbar pain persisting for one month, lacking an identifiable cause and hematuria, while maintaining satisfactory mental and dietary conditions. External CT imaging revealed a massive cystic lesion in the right upper abdomen, originating from the right kidney, suggestive of severe right renal hydronephrosis. The patient was admitted to our hospital for further evaluation and treatment. Upon admission, he reported a history of fracture surgery with no infectious or chronic diseases. A palpable mass was identified in the right upper abdomen; CT results revealed severe right renal hydronephrosis accompanied by multiple stones (**Figure 1A**). Consequently, the provisional diagnosis was severe right renal hydronephrosis, attributed to right ureteral stricture. Following the exclusion of surgical contraindications, an open right nephrectomy was conducted. Intraoperatively, exposure of the renal fascia revealed a significantly enlarged right kidney with thin cyst walls measuring approximately 20cm*15cm (**Figure 1B**), from which 1000 ml of pale yellow turbid fluid was drained. Pathological examination of the excised tumor revealed histological features indicative of a spindle cell tumor, including neutrophil infiltration in the right kidney (**Figures 1C, D**). Immunohistochemical staining showed positive vimentin, PAX8 in epithelial and occasional spindle cells, CK in epithelial cells, CK5/6 in epithelial cells, CD99, CD10, focally weak EMA, CD68 in histiocytic cells, 40% Ki67; negative SMA, Myogenin, ALK, CD34, MyoD1, Bcl-2, ETV6, S100, Desmin, and PAS (**Figure 2**). Based on the comprehensive pathology and immunohistochemistry findings, the diagnosis was confirmed as sarcomatoid renal cell carcinoma with local ossification and vascular invasion. The patient received immune-targeted therapy consisting of Atezolizumab (1200 mg intravenous infusion on day 1) and Bevacizumab (600 mg intravenous infusion on day 1) two months post-surgery, followed by a second round of immune-targeted therapy in the third month post-surgery. Subsequently, the patient underwent regular follow-up examinations. As of the manuscript revision (15 months post-surgery), the patient remains alive.

## Discussion

SRCC accounts for only 5% in RCC and represents a highly refractory and lethal form of kidney cancer (2). The most common symptoms among SRCC patients include pain (51–52%), hematuria (22–34%) and systemic symptoms (4), whereas cases of SRCC accompanied by severe renal hydronephrosis are exceedingly rare. In 2012, Kimura R and colleagues (5) documented the first case of SRCC with severe renal hydronephrosis. In this case, a 53-year-old male patient sought medical attention for visible hematuria and left shoulder pain. CT imaging revealed an extensive cystic lesion in the left kidney, which led to a left radical nephrectomy. Subsequent pathological analysis confirmed a diagnosis of sarcomatoid renal cell carcinoma. Similarly, another patient presented with persistent lumbar pain of unidentified origin, and CT scans indicated severe renal hydronephrosis in the right kidney. Consequently, an open right nephrectomy was performed, revealing postoperative pathology consistent with sarcomatoid renal cell carcinoma. This case represents the second documented instance of SRCC associated with severe renal hydronephrosis. A systematic review of all SRCC cases published since 2014 can be found in **Table 1**.

**Table 1:** Literature review of published cases.

NO.	First Author	Age/Sex/Side	Clinical symptoms	preoperative diagnosis	surgical treatment	pathology	Transfer site	Stage	Adjuvant treatment	Follow-up (months)
1	Wu, et al	33/M/NR	Hypertensive emergency	TAA-RA; AAT	AATR	SRCC	AA-RA	NR	No	8/DOD
2	Nadine, et al	83/F/L	Renal mass and retroperitoneal adenopathy	RCC	ORNLD	CCPRCT; SRCC	LN	T3aN1M0	No	5/transfer to L2
3	Yu, et al	65/F/L	Intussusception and gastrointestinal bleeding	MIT	N	SRCC	SI	NR	thymosin	44/NED
4	Yuji, et al	53/M/R	Heat generation and Right renal pain	RCI	N	SRCC	LN	T1N1M1	No	15/DOD
5	ALEXANDRU, et al	79/M/L	Visual hematuria and Lower back pain	SRCC	N	SRCC	NT	NR	No	NR
6	Zhang, et al	64/F/R	Right upper abdominal pain and Oral ulcer pain for 6 months	RCC	N	SRCC	LV	NR	TKIs	NR
7	Amit, et al	58/M/L	Severe abdominal pain, Visual hematuria and Left renal mass	RCC	N	SRCC;PRCC	LN	NR	No	2/Metastasis/DOD
8	Liang, et al	45/M/R	gross hematuria	RCC	N	SRCC	RS-U-T3	T3N1M1	No	0.5/respiratory failure/DOD
9	Fuser, et al	63/M/NR	Left rib pain, severe dry cough and difficulty breathing with force	MPBLC	NO	SRCC	brain	NR	ABRAXAN, Pembrolizumab	DOD
10	Logunova, et al	73/F/NR	Upper back lump	RCC	N	SRCC	skin	NR	No	NR
11	Ahmad, et al	17/F/L	Lower back pain, hematuria, abdominal swelling	RCC	LCNE-LNE	SRCC	Liver, lungs, and spleen	T4N2M1	Adriamycin, vincristine	10/DOD
12	Yaegashi, et al	62/F/L	gross hematuria	RCC	N	SRCC	Bone and liver	T3N2M1	Interferon-α, axitinib, everolimus, radiotherapy	71/DOD
13	Bukelo, et al	55/M/NR	Lower back pain and hematuria	RCC	N	SRCC	lungs	NR	NO	NR
14	Huang, et al	36/F/L	abdominal pain	RCC	N	SRCC	NT	T2N1M0	NO	1.25/DOD
15	Huang, et al	56/M/R	Abdominal pain and fever	RCC	N	SRCC	NT	T2N0M0	NO	1.67/DOD

M, male; F, female; R, right; L, left; NR, no reported; TAA-RA, thrombosis of abdominal aorta and renal artery; AAT, abdominal aortic tumor; RCC, renal cell carcinoma; MIT, metastatic intestinal tumor; RCI, renal cyst infection; SRCC, sarcomatoid renal cell carcinoma; MPBLC, metastatic primary bronchogenic lung cancer; AATR, abdominal aortic thrombus removal surgery; ORNLD, Open radical nephrectomy and lymph node dissection; N, nephrectomy; LCNE-LNE, left cytoreductive nephrectomy and lymph node excision; AA-RA, Abdominal aorta and renal artery; LN, lymph node; SI, small intestine; NT, no transfer; LV, lymphatic vessels; RS-U-T3, Renal sinus, ureter and Third thoracic vertebra; TKIs, Tyrosine kinase inhibitors; DOD, died of disease.

The term “Giant hydronephrosis” (GH) describes a condition where the kidney’s collecting system holds over 1000 milliliters of urine or the kidney comprises at least 1.6% of total body weight (6). GH occurs more frequently in children than in adults. The primary congenital cause of GH is obstruction at the renal pelvis-ureter junction. Additional causes include ureteral ectopia, duplicated collecting systems, and abnormal vascular systems that compress at this junction, also leading to GH (7). Regarding the association between GH and SRCC, sarcomatoid tumors are typically large, averaging 9–10 cm in size (3). Consequently, the tumor’s presence can lead to local obstruction, potentially causing multiple stones in the right renal calyx and impaired urine flow, which may contribute to renal hydronephrosis. In our case, despite the use of CT imaging, distinguishing between severe renal hydronephrosis and sarcomatoid renal cell carcinoma remains challenging. Here we summarize the similarities and differences between SRCC and GH in **Table 2**.

**Table 2:** Similarities and differences between SRCC and GH.

	SRCC	GH
Clinical manifestations	pain, hematuria and systemic symptoms	Abdominal distension, nausea, back pain, and hematuria
Diagnostic tools	Pathology and CT	Urography, ultrasonography and CT
Pathological features	Spindle cells	Cellular and Interstitial Edema
Treatment	Nephrectomy, Chemotherapy and Immunotherapy	Nephrectomy or renal sparing therapy

The pattern and degree of pleomorphism do not influence clinical behavior; therefore, all SRCCs are classified as International Society of Urological Pathology grade 4, reflecting their poor prognosis (8). SRCC is highly invasive (1) and typically occurs in patients at advanced stages or who have already experienced metastasis (9). Numerous studies have confirmed that sarcomatoid transformation independently impacts patient prognosis, with more extensive transformations associated with poorer outcomes. Reports indicate that the median survival for SRCC patients typically ranges from 4.9 to 19 months (10–13). Presently, nephrectomy remains the predominant treatment strategy for SRCC. Michail et al. analyzed clinical data from 879 SRCC patients in the SEER database, finding that nephrectomy significantly improves patient outcomes. All patients eligible for surgery should undergo consideration for nephrectomy (14). Exploration of diverse and more effective treatment methods continues, with immunotherapy playing a pivotal role. A meta-analysis involving 467 patients suggests that immune combination therapy significantly reduces mortality and disease progression compared to sunitinib treatment (15). Robot-assisted partial nephrectomy (RAPN) is currently utilized in the treatment of renal cell carcinoma, showing promising results (16). Hanif’s retrospective study also shows that therapy with immune checkpoint inhibitors (CPI) offers superior outcomes compared to conventional treatments using molecular targeted drugs or chemotherapy (17). Therefore, immunotherapy presents a promising approach to potentially improve the prognosis of patients with SRCC.

In future clinical scenarios, it is crucial to recognize the potential for sarcomatoid renal cell carcinoma to present as significant renal edema, which may indicate a more aggressive pathological subtype and greater invasiveness.

## Conclusion

SRCC represents a highly invasive malignancy with a pessimistic prognosis within the urinary system. Clinicians must remain vigilant to identify the potential presence of sarcomatoid renal cell carcinoma in patients exhibiting clinical signs and imaging findings suggestive of renal hydronephrosis, thus preventing delays or misinterpretations in diagnosing SRCC.

## Data availability statement

The original contributions presented in the study are included in the article/supplementary material. Further inquiries can be directed to the corresponding authors.

## Ethics statement

Written informed consent was obtained from the individual(s) for the publication of any potentially identifiable images or data included in this article.

## Author contributions

SC: Writing – original draft. ZH: Writing – original draft. MM: Writing – review & editing. ZT: Writing – review & editing. CZ: Writing – review & editing. NF: Writing – review & editing. BY: Writing – review & editing.
